# Visible, invisible: Black women in higher education

**DOI:** 10.3389/fsoc.2023.974617

**Published:** 2023-04-20

**Authors:** Victoria Showunmi

**Affiliations:** Department of Education, Policy and Society, University College London, London, United Kingdom

**Keywords:** intersectionality, women, race, leadership, higher education

## Abstract

This paper explores race and gender in the context of higher education, analyzing the experiences of Black women in academia to create a better understanding of what it is to be Black and a woman in contemporary British society. The main themes of this paper are elaborated through the lens of critical Black feminism. The historical origins of inequalities are outlined foregrounding their influence on how Black women are described and regarded. The damaging impact of everyday and sophisticated racism intersecting with sexism is explored and exemplified. Concepts and theories serve to elucidate the discrimination suffered by Black women in academic roles. White women do not offer unequivocal support to their Black colleagues and may even undermine their career progression. When Black women gain leadership roles, the traditional characteristics associated with leaders often conflict with the stereotypical expectations of Black women. A closer examination of higher education reveals the extent of the racial trauma endured by Black women and the resultant decline in their wellbeing.

## Black women and inequality in the workplace

“Living life as black women requires wisdom because knowledge about the dynamics of intersecting oppressions has been essential to black women's survival… Black women cannot afford to be fools of any type for Other objectification as the Other denies us protections that white skin maleness and wealth confer.” Collins, 2002, p.257.

## Introduction

This paper explores race and gender in the context of higher education (as a workplace offering precarious part time and sometimes full-time employment), analyzing the inequality experienced by Black women to create a better understanding of what it is to be Black and a woman in contemporary British society. The main themes of this paper are elaborated through the lens of critical Black feminism.

This is a review paper using a conceptual approach. It presents an overview of key current issues of inequality which emerge through sophisticated and everyday racism. This article focuses on the context of academia and draws on extensive research carried out between 2012 and 2016. Successive iterations of empirical research have informed the development of the concepts (sophisticated racism, white women syndrome), which are detailed here. This is not an ethnographic research paper in itself.

Fundamental concepts, examples and a case study confirm that inequalities have been reconstituted in new forms. Issues and problems related to misconceptions including notions of a “post-racial” era are analyzed. The conditionality of equality gains achieved so far is revealed. Examples of gains claimed in reports (Commission on Race and Ethnic Disparities, 2021) are deconstructed, highlighting the uncertainty of fictitious attainments. There may be legislation, but equality has not yet been achieved, especially for Black women. Black women are experiencing completely unacceptable racism at work which has become normalized (Fawcett 25th May, 2022).

Concerns relating to workplace inequalities are foregrounded. Everyday and sophisticated racism combined with sexism has a damaging impact (Showunmi and Tomlin, 2022). The dominance of White women and their role in discrimination against Black women is discussed (Showunmi and Tomlin, 2022). A fresh cause of inequality is White Women Syndrome (WWS) (Showunmi and Tomlin, 2022). WWS is a term that has been coined by Showunmi to describe the often hidden behavior of some white women in the workplace and wider society. The “pet to threat” (Thomas et al., 2013) phenomenon whereby support is initially provided then withdrawn when Black women progress in their careers is detrimental to advancement. The historical roots of racism in the workplace, especially as experienced by Black women, are uncovered, and the evolution of the term “Black” and its significance in everyday usage in the workplace is also central. The persistence of racism despite notions of a post-racist society is elucidated through the theory of sophisticated racism (Showunmi and Tomlin, 2022).

## Literature on Black women in the workplace

White women academics have written extensively on women's position in the labor market with a focus on gender and the division of labor. They have emphasized the disadvantage to women caused by patriarchal power (Cockburn, 1981; Walby, 2000; Saguy and Rees, 2021). Ethnic minority women are rarely included in these analyses and when they are, the analysis tends to focus on the “double disadvantage” of sexism and racism. White women (researchers or organizational leaders) have seldom reflected on their own ethnic privilege including how this has impacted on their work, MacIntosh (1989) being a notable exception.

Research carried out by Erete et al. (2021, cited in Hampton, 2021) noted that there was an upsetting theme that appeared in the interviews she conducted on the leadership journeys of Black women in academia. The interviewees all mentioned that at the beginning of their careers they believed they were very well liked and almost overprotected by colleagues. They were aware that these positive attitudes emanated partly from the recognition that they were a token Black woman who was employed to prove that the organization was inclusive and complying with equality legislation.

However, as they started to push forward in the organization and seek opportunities to develop further the “benevolent tolerance they enjoyed transformed into pushback, if not outright hostility”. Thomas named this as the “pet to threat” phenomenon. The hostility appeared to manifest itself when the Black women were in line for promotion or leadership roles.

These experiences result in justified paranoia and hypervigilance, which adds to the burden of the “double consciousness” described by Du Bois (1999). This is an added challenge which Black women in academia endure on a regular basis.

Cultural difference is an area which is difficult to pin down as discussed by Showunmi et al. (2016) in her research on leadership and identity. Unspoken cultural norms can create the sense that colleagues come from a different planet. The hypervisibility that comes with being “the only one” can lead to White employees constantly second-guessing Black women's instincts. This questioning attitude can lead to Black women suffering in silence and can exert a negative impact on their wellbeing (Showunmi and Maylor, 2013).

Within predominantly White and male dominated universities, the traditional characteristics associated with leadership often conflict with the stereotypical expectations of Black women (Parker, 2004; Rosette et al., 2016). For example, Black women's communication style is often stereotyped as blunt, which is consistent with a Eurocentric view of masculine communication as direct and controlling (Olasunkanmi-Alimi et al., 2022).

The resultant dearth of Black women in senior positions applies across tertiary education. Between 1997 and 2006, almost all Vice-Chancellors (VCs) appointed were white, 23% had studied at Oxford or Cambridge universities, and 85% were male (Breakwell and Tytherleigh, 2008).

Until 2011 there had only ever been one VC from a black and minority ethnic background–a male, non-UK national (Burkinshaw, 2015). However, by 2013, there had been three new BME VC appointments, two female and one male in England. The representation of Black academic staff in senior positions remains low. Of almost 23,000 UK professors, only 41 are Black women (When Equality, 2023).

Bhopal and Chapman (2019) in her paper states that BME groups continue to be marginalized in HEIs; they are underrepresented in the highest contract levels and overrepresented in the lowest, they are less likely to occupy senior managerial positions, less likely to be professors, and less likely to be on the highest pay band compared to their White colleagues. Within the BME category it is Black groups who continue to suffer the greatest disadvantages at all levels. The pattern of hierarchical segregation across gender and racial/ethnic lines encountered in higher education careers is repeated in the other spheres such as politics and prominent business organizations.

Often, the only way for women to distinguish themselves in the workplace is based not so much on what they say but what they wear and most importantly how they look. This is what Wolf (2002) refers to as the Professional Beauty Qualification (PBQ). This PBQ is based on the premise that “all the professions into which women are making strides are being rapidly reclassified”.–so far as the women are concerned–as display professions. Wolf suggests that prior to women entering the workplace, there was a clearly defined class of women explicitly employed for their “beauty”. Such jobs included fashion mannequins, dancers, and higher paid sex workers such as escorts; it has now been extended to include flight attendants, actresses and secretaries. PBQ is being widely institutionalized as a condition for women's hiring and promotion. PBQ can lead to ongoing harassment and even for women to lose their jobs once they no longer look the part. Abass continues with the argument that it is a reality that beauty has become a “legitimate” and necessary qualification for a woman's rise in power.

Abass (2008) suggests: “there is no such standard for men. Although men are expected to conform to a standard that is well groomed and often uniformly clothed, women are to dress in a way that is not only professional, but also conforms to an institutionalized standard of beauty” (p. 40).

Whilst there have been improvements and increased opportunities for women across higher education, it remains clear that access to the top table is not available to them. The stress and racial burnout experienced by many Black women in the academy emerge in much of the literature. Burgis (2009) study examined the experiences and perceptions of Black women. It reflected on how the work experiences of Black women are affected by the factors of age, race, and gender.

The assumptions within feminist theories and Black feminist theories that are sensitive to the realities and contextualized lives of this population were discussed. The findings of this research suggest that while Black women perceive they are fulfilled and successful at work, they still struggle with racial and gender stereotyping, workplace discrimination, and inter-racial resentments. Hall et al. (2012) explored work-related stressors that affected the lives of Black women and how they coped with them. Using an exploratory design with grounded-theory methods, five basic themes emerged that identify when racism and sexism are experienced as stressors for African American women in the workplace. The themes are: (1) being hired or promoted in the workplace, (2) defending one's race and lack of mentorship, (3) shifting or code switching to overcome barriers to employment, (4) coping with racism and discrimination, and (5) being isolated and/or excluded. The results from this study indicate African American women use emotion- and problem-focused coping responses to manage stress (e.g., racism and sexism). Holder et al. (2015) study experiences of racial microaggressions in the workplace and the coping strategies of Black women managers, recounting the struggles that Black women face. Black women feel compelled to shift their identities to mitigate the negative outcomes associated with discrimination.

Writing on “race”, particularly in relation to the education of Black men (not women) in the British literature, tends to be undertaken by White male academics (for example, Ghaill, 1988, 1996; Gillborn, 1990, 2008). More recently White women (Archer and Francis, 2006; Archer, 2011) have also started writing about issues of “race”. It is unlikely that Archer and Francis have personally experienced racialized encounters, which Black women often face on a daily basis. Despite the insightful work carried out by some White writers, in some ways, they have colonized and capitalized on race and diversity issues, so much so that Ahmed (2012) considers that the production of these works has become an industry. Discourses and scholarship on gender and race continue to be dominated by western frames.

## Historical origins of the exclusion of Black women

The exclusion of Black people from the workplace has been part of British history. If we question why and how Black people were brought to the UK, that in itself is interesting. Originally (18th century), most came to enter domestic service for the upper middle classes. This consisted of chores such as cleaning, ironing, and gardening. Once they had learnt those skills, Black people were used as accessories as they looked exotic by the side of their employers. Bringing a Black person to the UK implied that the Black employee was akin to a pet kept by its owner. Some were educated at the employer's expense. Others could learn a craft alongside their other duties. All this was on the employer's terms and with their agreement. Some employers used to give away their Black employees, their “prized possessions”, on condition that if the recipient found they did not like the “gift”, they would hand them on to another employer. All the above is relevant as the low status of the first Black women to come to the UK connects to the present-day workplace.

There has been a long history of the exclusion of the experiences and voice of Black women. However, despite this Black British women's voices continue to be abandoned in the wilderness. The authors of *Heart of the race* (1984) were the only writers brave enough to enter into a wide-ranging discussion including topics such education, work, health, and issues in the community.

Interweaving the past with the present, recognizing the strong influence of the historical experiences of Black women, enables us to remember and better understand the importance of the role played by our foremothers. The concept of the “double day” (Bryan, 2018) was an established feature in the lives of Black women before they arrived in Britain. In practice, despite their inferior status, there has been no distinction between Black men's and Black women's labor, as women have been expected to work just as hard and for as long hours as Black men. Black women acquired the reputation of being strong and were seen as better investments for employers as their life expectancy was 5–6 years longer than that of Black men.

The misrepresentation of Black Girls in school has led to a vast number being excluded and or facing mental health issues as part of being a young Black girl in society. The lack of understanding of Black girls by educators begins from the tender age of six. This has been documented by researchers such as Morris (2007) who speaks about the unnoticed and silent voice of Black girls. In order to fit into the dominant culture, many Black girls have learnt the art of dumbing down or “shrinking” to ensure that they are able to fit in without being noticed (Sue, 2005).

The art of dumbing down runs counter to the ethos in higher education. Anyone aspiring to climb into the ivory tower, especially in the role of an academic, must be able to hold their own in intellectually demanding contexts. Policy has attempted to pave the way for the participation of more Black people in higher education. Initial participation is naturally the first step toward gaining an academic post.

Digital Education Resource Archive (DERA) (2002) and the Equality Act 2010 (2010) both required higher education institutions (HEIs) to implement equality policies which demonstrate their commitment to race equality. In order to address gender inequalities in higher education, the Athena SWAN charter was introduced in 2005 by the Equality Challenge Unit to improve the position of women in STEM subjects. With growing evidence of racial inequalities in HEIs, in 2014, the Race Equality Charter (REC) was introduced to address student attainment, staff progression and the curriculum (see Bhopal and Jackson, 2013). The REC ‘… provides a framework through which institutions work to identify and self-reflect on institutional and cultural barriers standing in the way of minority ethnic staff and students' (https://www.ecu.ac.uk/equality-charters/race-equality-charter/about-race-equality-charter/).

Instead of attaining prestigious roles in the academy, Black women have often been forced to fill the jobs which the dominant workforce is no longer willing to do, in the service industries, the semi-skilled and unskilled sector. When they manage to move into other sectors, they are seen as “trouble-makers” if they stand up for better conditions and the rights of women in the workplace. In higher education, “women, Black staff, and disabled staff continue to face shocking pay gaps, with women more likely to be on zero-hours contracts than men, and black women even more likely still” (UCU General Secretary, February, 2022).

In the workplace, initially at the interview, Black women are wanted. Post arrival, white employers do not know how to welcome and encourage the Black women employees and are unable to integrate them into the workforce. As a result, the new Black employee starts to wither. No one supports them. They lose confidence and become excluded (Cokley et al., 2011).

The exclusion of Black women has been justified extensively so that it has become challenging to pinpoint the precise source of current racism. It is sophisticated and complex with many layers. When one layer is removed, another is uncovered, revealing more interconnected threads.

## Black women and unemployment

“Unemployment rate measures the ratio of people looking for work as a proportion of all those able to work; otherwise known as economically active. People who are unemployed are those who say they want to work and are available to work and have looked for work” (Shire, 2022).

Statistics have shown that Black women in the UK suffer from higher rates of unemployment than White women. The writes: “White women (3%) were less likely to be unemployed than women from all other ethnic groups combined (7%)”.

On the above definition, the average unemployment rate for Black people in the UK has remained at about twice that for whites for both sexes from as far back as when the statistics on race were first published in the 1991 census. Although rates of unemployment for women have tended to be marginally lower than those for men, the data disguises the fact that women are more likely to seek part time work or are discouraged from looking because of a lack of suitable employment where say childcare is an issue. Nevertheless, Black women unemployment rates are at about 8% compared with about 4% for White women. The gap in unemployment between the ethnic groups seldom changes; tending to rise and fall in parallel. The gap in employment rates tends to be greatest at under 25 age groups. The latest unemployment for the year to June 2021 for London shows that whilst the rate for Whites aged 16 to 24 is 18.2%; that for Black people in the same age group is 37.4% (Shire, 2022).

Research has revealed a range of issues that may be preventing Black women from achieving their full potential. However, there is almost no research which explores and analyses the experiences of unemployed Black women (Showunmi, 2009) and any barriers that may be preventing them from succeeding in gaining employment.

In general, much of the data on the situation of Black women stems from qualitative rather than quantitative methods. Some quantitative researchers may reject the evidence generated by small scale qualitative studies.

Showunmi's (2009) study examined some of these issues to enhance understanding of the obstacles encountered by unemployed Black women attempting to access education, training, and employment. A qualitative ethnographic approach was adopted. Data was gathered through interviews and interactive groups with participants who were unemployed Black women aged between 17 and 45 in Southeast England. The research revealed the complexity of race, gender, and identity in this context.

The findings identified key issues that appear to be hindering the success of Black women in education and employment. These issues revolve around direct and indirect racial discrimination, culture, identity, and a lack of racial and cultural sensitivity among educators and employers. The study showed that an important reason why some unemployed Black women do not engage in education or employment is related to their sense of who they are. They constantly have to grapple with adapting to the contemporary, white-dominated workplace, whilst their authentic self is rooted in a culture which emanates from a distant family heritage.

Perhaps if they knew and understood their own identity, they would feel more confident to reach out to take advantage of the opportunities on offer. The following quote highlights this;

I have lived here all of my life and I am concerned that I have to define my own background…when I constantly keep being asked who I am and where do I belong? (Interview participant, Showunmi, 2012).

Answering persistent questions about your origin adds to the emotional and racial burden which is carried into the workplace. It creates an ongoing narrative, suggesting it is acceptable to keep asking questions regarding who you are when it is obvious you are third generation in the country.

More black women are graduating with advanced degrees than ever before (Bhopal and Chapman, 2019). If they manage to secure employment in higher education, they find themselves employed as casual faculty on precarious contracts (Nzinga, 2020). Despite their advanced educational and potential professional opportunities, highly educated women are facing worsening economic hardship and poor working conditions at universities. They are frequently on short-term contracts which prevent them from repaying high levels of student debt. Their contribution to the intellectual life of their disciplines and departments remains unrecognized. They are burdened with heavy teaching workloads which prevent them from making progress with their research (Showunmi and Maylor, 2013).

## Blackness, Black women, and feminism

Black women find it challenging to accept the term “feminism” because it is inextricably linked to White feminism (Syed and Ali, 2011). Some Black women therefore prefer the term “womanist”, coined by Walker (2011). In relation to Black women, it is argued by scholars such as Collins (1999) that one of the unwitting consequences of the increased accessibility of knowledge about the lives of Black women is that they are objectified rather than being subjects of investigation. Collins (1999) suggests that a process of “knowing without knowing” occurs, whereby dominant groups in society who have a particular interest in understanding the lives of Black women can observe them from a distance, without having to personally interact or critically engage with them. Such investigators do not have to critically analyse their own lives. In contrast, the racial and gender status of Black women means that they are not afforded the opportunity to view other social groups objectively because any understanding of their own lives is interpersonal and based on their status as the racialised and gendered “other” (Reynolds, 2002).

Critical Black feminism shapes the theoretical framework of this article. Black feminism is defined as a way in which women who are Black collectively think and become as one. It is a philosophy that centers on the idea that “Black women are inherently valuable, not as adjuncts to somebody else but as human beings in their own right”. Black women's liberation is a necessity. Black feminism centers on the experiences of Black women, understanding their position in relation to racism, sexism, and classism, as well as other social and political identities. Black women have been excluded from mainstream feminism because of their race, while simultaneously being excluded from black liberation movements because of their gender (Collins, 2002).

Black feminism highlights and engages with the many aspects of identity that women have, including their professional identity as Black academics, which is significant because it gives them the opportunity to talk about being Black parallel to gender inequality (Collins, 2002; Hooks, 2014). Despite the prejudice and discrimination they faced, Black women were and continue to be critical to the black liberation and gender equality movements.

Black feminism advocates women's emancipation and empowerment; Marxist social thought aims for a more equitable society, while queer theory opposes heterosexism. Women who identify as migrants find it difficult to identify with mainstream feminism and find their way to Black feminist movements. Black women who identify as feminists are automatically connected to radical feminism. Black women such as Kathleen Cleaver, Angela Davis, Assata Shakur and Claudia Jones were seen as Black radical feminist intellectuals and activists. Before this was Sojourner Truth, Harriet Tubman, and Rosa Parks (who used silence as a weapon for change).

The labeling of Black women as radical feminists projects a particular challenge as it is fuelled and driven by the political stance of wanting to belong and be included on their own terms. Black women in the academy can be regarded as disruptive (Hooks, 2014). Much of the feminist movement has been formed on the back of institutional reforms which aim to reduce gender discrimination and give women access to male dominated spaces, promoting gender equality. Black women have produced social thought designed to oppose oppression. Interestingly, this form of thought does not just rely on traditional academic writing, as it opens a creative space in which to explore poetry, music, dance and art as a way of expressing Black feminism. Black feminism is an expression which has enabled women to find ways to survive in or oppose prevailing social and economic injustice. It becomes a way of life that is difficult to explain to people who are not Black.

In the early 1960s, being Black was a taboo subject. Blackness and Black identities were not discussed. In the UK, Black people were regarded as temporary visitors, immigrants who would return “home”, not as permanent members of society.

Blackness was downplayed, from the White perspective in the sense that what you don't name can be more easily ignored. Black people were expected to be “seen and not heard”. This led the Black population to feel invisible. Humility and eternal gratefulness for the right to migrate to the UK were expected from the host country. This feeling of gratitude became ingrained in the Black psyche and underpinned the mindset of many Black communities (Pheterson, 1986).

Any Black individuals who adopted a different mindset were regarded as alien and would experience a difficult time in the workplace and the wider society. The resultant silence of Black women is highlighted by Bryan (2018), who were the first writers to give a voice to Black women in the UK, correcting misleading versions of British history which excluded them. Black women who described their experience of marginalization and discrimination did not fit the passive mold which had been shaped for Black people. This was particularly relevant in the workplace, as it was regarded as a privilege to be “invited” to join the majority White workforce.

## Terminologies of Blackness

I now examine how the word “Black” has emerged both in the workplace and more widely. The term “Black” came into use after the Civil Rights Movement in the United States during which people who were not white united as one. The epithet “Black” also encompassed other characteristics associated with Blackness beyond skin tone. Hair, dress and even ways of speaking were identified as an integral and distinctive part of the Black identity.

It is important to note that there is a lot of discussion about the use of terms across the globe, including the “correct” way to refer to people who are not white. The discussion which follows aims to reveal the complexity of these issues. The identification of the acceptance of specific terms will add to the literature in this area. People who are racialized as non-white have been forced to grapple with a range of terms to describe who they are, terms which have often been developed by people who are not Black.

To complicate the situation further, the terms used depend on the country where the person who is racialized as non-white lives. The terms include Black Minority Ethnic (BME), Black and or Asian people, Non-white, Black Indigenous People of Color (BIPOC). ethnic minorities, Africans, African Americans, African–Caribbean, Black Europeans, Negro and Migrant. Historical context is necessary for clarification.

On the surface, these terms may appear to have emerged from the people who are themselves racialized as non-white. This is not the case. In the main, many of the terms included in this list have been used as labels to identify people who are racialized as non-white without consultation. So why does this matter? Well, if you are White then you are just White no matter where you are in the world. There is simply acceptance, and no further discussion takes place. However, when for example a person migrates from South America to the United Kingdom, they may automatically assume that they are white, but this is where the complexity begins to manifest itself. There are many layers which complicate how people who are racialized as non-white are seen in society.

It is not until the South American arrives in the UK that they are repeatedly asked “where are you from?”. The question could of course be prompted by a non-British accent. In actual fact, the South American is more likely to be experiencing a skin- tone challenge. The question stems from a darker skin tone and/or wide-ranging cultural and behavioral attributes which are not associated with whiteness, particularly “Englishness”. The skin-tone challenge would create a ripple effect of successive waves of unease by underlining the immigrant's sense of “Otherness” in their new environment. They may have been privileged in their country of origin, but migration has deprived them of their identity. They are simply excluded outsiders who do not belong in a Britain which is hostile to migrants.

The consideration of how people are identified when leaving their country of origin depends on layer upon layer of factors akin to the layers of an onion. These layers would include their socio-economic status, cultural capital and individual attributes, class, language, and skin tone. All but one of these fits into Bourdieu's concept of “Habitus”, (Bourdieu, 1993) which refers to the norms, values, attitudes, and behaviors of a particular social group (or social class). Skin tone is not included but has always been the elephant in the room. It is too uncomfortable to acknowledge that employers and colleagues associate darker Black skin tones with inferiority and therefore a poorer performance at work. These views originated from eugenics, which were considered scientifically sound and were believed and respected in the 19th and 20th centuries (Galton, 1909). These beliefs have resurfaced and been exacerbated by relatively recent far right discourses. Murray and Herrnstein (1994) triggered *The Bell Curve* debate based on the unsubstantiated contention that darker skin correlates with lower intelligence. In 2011, Kanazawa, a London School of Economics (LSE) academic, maintained that data showed that Black women were the most unattractive in the world. He later admitted that his “race” analysis was flawed. Kanazawa is not alone in using flawed data to suggest that Black people are inferior to white people.

Wade et al. (2004) explored how skin played a role in the hiring of employees. It was found that Black women with lighter skin colors were given better privileges than those with darker skin tones. In addition to this Neal and Wilson (1989) research revealed further concerns. Skin color, facial features, and hair play a significant albeit subtle role in the lives of Black Americans. For many Black Americans, central feelings related to perceived self-worth, intelligence, success, and attractiveness are determined by such factors as the lightness of their skin, the broadness of their nose, the thickness of their lips, and the frizziness of their hair. Although these characteristics affect both men and women, psychologically these effects appear to be stronger for females who, regardless of race, have had to concern themselves more with appearance.

The ranking of color in relation to privilege gained momentum when Black people were enslaved across the globe. In the hierarchy of the shades of skin color that emerged, enslavers did not officially recognize their mixed parentage children (European and African descent for example), but they nevertheless accorded them privileges that dark-skinned enslaved people did not enjoy. Consequently, light skin came to be regarded as an asset in the community of enslaved people.

A pyramid of white privilege emerged. White was at the top, Asian was in the middle and Black African was at the base. This persists in the academy today. The more you resembled a white European, the more benefits you accrued. The pyramid illustrates the notion of “Colourism”. Reece (2016) and Feliciano (2016) argue that colourism is a system of practices and ideologies that privilege lighter-skinned Black people, with facial features typically associated with Europeans, over their darker-skinned counterparts with facial features which are generally seen to be characteristic of Africans.

Such entrenched practices are believed to have disappeared, but they persist in the form of what I have called “sophisticated racism”. Sophisticated racism is a smokescreen which hides racist epistemologies using complex ideals couched in liberal ideology. I have defined sophisticated racism as follows:

Sophisticated racism is based on systemic structures designed to promote racism while disingenuously promoting anti-racist or equitable policies. The perpetrators would not want to be accused of racist behaviors and they pay lip service to condemning racism. Sometimes this form of racism is unconscious and subconscious (Showunmi and Tomlin, 2022).

The impact of mental schemata is important here as this represents how events are recalled in the mind. Mental schemata are shaped by a person's political and cultural background. When used in the workplace, they shape and disguise racist behaviors. They convince the perpetrators of sophisticated racism that their actions are intended to be beneficent. They find themselves saying:

“I didn't know, I was just trying to help.” Or “it's not for us” (meaning white people)

The above illustrates how they attempt to distance themselves from their sophisticated and everyday racism. They are thoughtless and oblivious of the impact of their behavior.

There is evidence that colourism affects the amount you earn, lowers marriage rates, and leads to longer prison terms and fewer job prospects for darker skinned people who live in countries where the population includes people of European descent (Tomlin, 1999; Maylor, 2014). Colourism is not only persistent, but one of the most pervasive expressions of racism. As it results in poorer life chances for specific groups of people, it leads to discrimination which should be fought as a matter of urgency.

## The relevance of colourism in the workplace

Colourism is relevant because it is fundamental to how Black women are perceived in the workplace (Abass, 2008; Gabrielle, 2008). Notions of colourism may appear to run counter to what is perceived as contemporary British society where racism is supposedly a thing of the past. The belief that we live in a post-racial society now that there has been a Black President in the White House is widespread (see for example the report of the Commission on Race and Ethnic Disparities, 2021).

However, the true situation is that in many respects, we are living in a “new Jim Crow” era (Pilgrim, 2000). Pilgrim details the profound underpinning of the US “racial caste system between 1877 and the mid-1960s”, describing it as “a way of life” whereby African Americans were second class citizens and anti-black racism was legitimized, including by academics and Christian theologians. Blacks were regarded as “innately intellectually and culturally inferior to whites”. Black people were frequently subjected to violence including lynching if they attempted to question their inferior status.

It is more comfortable for Black communities to support the new supposedly post- racial neoliberal order than to speak out and acknowledge the dangers of meeting racism with a wall of silence. It is alarming that even in the face of widespread evidence of deeply embedded institutional and individual racism, the recognition of racial division has become marginalized in economic policies and civil society (for recent examples of racism, see Hansard, 2021; Ungoed-Thomas, 2021 “Racism in Cricket” 9 November 2021). It must be pointed out that the examples here relate solely to the experience of men. Women are seldom invited to provide their evidence as Azeem Rafiq was.

The everyday struggles faced by Black women as a result of subtle and sophisticated racism are ignored. It is almost as if women who belong to Black minorities are so marginalized that they are forgotten. It is as though they do not exist.

## Black women in the workforce

Despite the many changes in the workforce, the residual sense of peripheral participation in economic activity remains in the Black population. This is particularly true for women. Today, organizations in western countries recognize that women make a significant contribution to their operations (Stockemer and Byrne, 2012).

Women's participation in the workforce has increased considerably since the mid- 1970s (Gregory and Connolly, 2007).

The Race Relations (Amendment) Act 2000 established good practice by making it a “general duty” for all public employers to promote race equality, consulting minority ethnic communities on policy and monitoring impact through race equality impact assessments from 2006. Employers now have clearly defined responsibilities toward their employees including their Black employees under equality acts, most recently the Equality Act 2010 (2010). The Act provides a legal framework to protect the rights of individuals and to advance equality of opportunity for all.

These initiatives were designed to lessen the adverse impacts of racism in the workplace. However, given the is a risk that any positive influence they have had could be weakened by notions that we now live in a post-racist society. The Commission on Race and Ethnic Disparities (2021) wrote a report which ignores the impact of institutional racism in the lives of young people. The Commission reached the controversial conclusion that institutional racism does not exist in the UK. Many spoke out angrily, suggesting that the purpose of the report was to whitewash the problems of racism in Britain. The report seeks to divide and rule, suggesting that some minorities do well whilst others fare far less well. The only minorities which are recognized are “model minorities” which refers to a minority group or a member of such a group stereotypically viewed as being more successful than other such groups or individuals.

The term model minority was introduced by sociologist William Peterson in his 1966 article in The New York Times Magazine about the success story of Japanese Americans in the US. In the article, Peterson makes reference to the fact that Japanese Americans, despite facing intense racism and discrimination, were still able to achieve success—in the way that other minority groups had not. Both the term and concept continue to contribute to what is being called the model minority myth, which is the false yet pervasive idea that all Asian Americans are equally economically and socially successful.

When the long awaiting response to the Commission Report emerged in March 2022, the Minister of State for Equalities did her best to provide a response which was intended to come from the heart as she was a Black woman. However, the response added fuel to an ongoing fire. The Commission failed to deliver on nine of its 10 key objectives. It does, however, essentially portray Black people as the authors of their own misfortune.

The reason for continued racism is Black people's supposed cultural dysfunctionality, lack of knowledge, understanding, unwillingness to integrate and adopt British values or any combination of the above.

The report recommends for example that school children be taught how enslaved Africans in the Caribbean culturally transformed themselves. The aim is to provide a more positive impression of the experiences of enslavement and colonialism, downplaying the suffering endured by slaves and failing to acknowledge the profits made at their expense.

Given the content of the report, it is not surprising that such a denial of racism will have an impact on everyday life including the workplace.

## The workplace

I define the workplace as a place of either part time or full-time employment. The Universities are the focus here as they are organizations which employ a diverse workforce to deliver education and create new knowledge. What is of interest is what it means to have a diverse workplace.

There are reams of policies and procedures designed to address the hostile environment which many Black women encounter in the workplace including in higher education. These are meant to make the workplace equitable, creating opportunities for Black women, and becoming richer and more inclusive than a space where only white people can feel welcome and enjoy career progression. However, those who benefit are White women, not Black women (UCU General Secretary, February, 2022). It is White women who have become the face of diversity. The success of White women at the expense of Black women is described in Showunmi et al. (2022) in relation to “White Women Syndrome” (WWS).

WWS is the unnamed feeling many Black women experience in relation to White women if they, as Black women, are getting too close to the “pot of gold”, recognition and reward for success. Only limited resources are available, and these have been earmarked for White women only.

Recognizing this helps to position the way everyday racism can be overlooked. Unexplained insidious behavior often originates from this and elements of unwelcome behavior come together here to generate a strong force field which results in sophisticated racism.

A well-functioning workplace team has the potential to create a platform for equal opportunities for all employees. Leaders want the workforce to accept the premise of embracing diversity. However, there are times when a team leader's theoretical wish for an effective diverse team cannot be realized. Team members find themselves unable to contribute to the smooth operation of their diverse team, in universities conducting research and delivering education. The reason for this is that fundamentally that would mean making sacrifices and some individuals in the team may not be ready to give anything up for the benefit of those who do not look like them.

Universities seek to meet the criteria embedded in the Race Equality Charter. However, when it comes to implementation, it is not possible to achieve the goals of equality in career progression in practice because of institutional failure to adhere to the standards which are espoused in theory but not in practice (Pilkington, 2011; Rollock, 2021).

Workplaces can engender the formation of distinctive groups of employees. These have been set up to harness difference to enhance inclusiveness, but paradoxically they can become divisive. Some employees with certain characteristics (LGBTQI, gender, race, disability etc) are eligible to join. Such groupings enable certain individuals to belong while others are left out in the cold because they may not fit into such neatly delimited categories. These groupings may actually add to the complexity of belonging.

## Identity: Intersectionality, stereotyping, and neoliberalism

It is helpful to consider the complexity of identity and categorization by considering the meaning of the term “intersectionality”, examined by Showunmi (2020). The term is frequently identified with critical race theory scholar Crenshaw (1989) who, along with other scholars, contributed to and advocated thinking critically about the multidimensional aspect of women's oppression along race, class and gender lines. According to Bernal (2002, p. 116) focusing on the intersection of oppression is vital because “one's identity is not based on the social construction of race but rather is multidimensional and intersects with various experiences”. Many argue that scholars using the “intersectional approach” will socially locate individuals in the context of their “real lives” (Weber and Fore, 2007, p. 123). Intersectional discussions examine how both formal and informal systems of power are deployed, maintained and reinforced through notions of race, class and gender (Collins, 1998; Weber and Fore, 2007).

There has been some attention given to Black women's experiences in the workplace including in higher education: Forson (2013), Healy et al. (2011). However, more needs to be done. Researchers still argue that sample numbers are too small hence the focus on statistical data is on men and women without using an intersectional lens. This creates an ongoing problem as the experiences of Black women are silenced and made invisible. Women not in the diaspora (in Black majority societies such as Nigeria and Ghana) have made great progress in the professions (Healy et al., 2004; Rodriguez and Scurry, 2019). Instead, there has been a genuine misunderstanding that these women are doing well and require no additional support or assistance. One could argue that such a belief stems from the works of feminist writers such as Mirza (1992, 2013); Mirza and Reay (2000) who maintains that Black women's resilience and strength have enabled them to persevere.

Black women's difficulties start during their schooling (Weekes, 2002; Bhopal and Maylor, 2014; Johnson and Ginsberg, 2015). Black girls face many challenges throughout their education. Research into their wellbeing (Showunmi, 2009) reveals disturbing experiences such as constant profiling as they progress through the school system, including interventions which prescribe how they should behave if they are to prosper in society.

There is much evidence to suggest that the hidden curriculum alongside societal norms have made Black girls “dumb down” and appear less intelligent than they are (Gatto, 2002; Leathwood, 2013). The stereotypical expectation is that Black girls are loud and disruptive (Fordham, 1993; Koonce, 2012). To counter the potential for disorder, they are forced into a straitjacket of limitations which silences them. This drains them of their energy, drive, and passion. Black girls may also develop low self-esteem and self-hate because of repeated efforts to restrain them. Similar patterns of belief and behavior toward Black women play out in the workplace.

Austerity and the cost of living crisis have contributed to the deterioration of the economic security of women of color. The frame of austerity actually distorts the experiences of poverty and economic inequality suffered by women of color. Well before the 2008 crisis, women of color were already living in a state of austerity; minority unemployment has consistently remained higher than for white people since records began.

The labor market experiences of Black women in Britain are particularly damaging. Where women of color are employed, they are more likely to be employed in low skilled, low paid and temporary work. A 2021 Trade Union Congress report stated that ‘women of color are almost twice as likely than white men to be on zero-hours contracts … [trapping] women in low pay and insecure work, unable to plan their lives and futures' (Institute of Race Relations, 2020). This translates into high levels of household poverty. 2007 poverty rates for minority groups were 40%, double the rate of white households. These shockingly high levels of poverty and unemployment pre-date the 2008 economic crisis and persist to this day yet seldom feature in the media or policy discussions.

Being Black and a woman in employment means fighting for recognition. It is not enough to have been chosen for gainful employment, Black women also need to prove themselves. This constitutes an emotional burden which many are not prepared for. This burden is invisible and is only seen by those who understand what it is and what it means to be Black and a woman in the workplace (Curtis and Showunmi, 2019).

In higher education, barriers to progression are considerable. Research (Showunmi and Maylor, 2013) revealed participants' awareness of their abilities and talents, which were not appreciated in the academy. One interviewee explained:

“I am frustrated because I feel that I have got the vision and I can see where I need to be but because I have been suppressed in my organization. I am unable to progress at the moment, so you know, I have got this vision and I know that it is a journey I am on, it is just to get there.” (Black academic)

Neoliberal policy and procedures protect the leaders of the establishment. Ahmed (2012) argues that a commitment to diversity is frequently substituted for a commitment to actual change. Ahmed traces the impact of the concept of diversity by examining how the term is used and how it makes questions about racism seem impertinent. Her research focuses on universities in the UK, Asia, and Australia, but her conclusions are relevant across the globe and in different settings.

Belief in policies on equality is widespread, but implementation rarely achieves the outcomes which underpin the visions and strategies of the universities. Equal opportunities policies are purportedly designed to provide equity for diverse groups in the academy. There is however much evidence (Dardanoni et al., 2006) that they have not been successful in achieving their purpose. Showunmi and Maylor's (2013) research confirmed this. One respondent stated:

I think that when I first set out, I thought I was going to be equal and be treated as everyone else, but I have kind of got used to it. I think I have realized over time; you know, you have to understand and work through disadvantage we experience as BAME women, and our managers have to acknowledge this too (Black woman academic).

There is a basic hierarchical structure at play in the typical workplace. There is a central leader and an inner circle which filters the decisions of the leader before they impact on the people who deliver the work. What is it like to become a member of that inner circle if you're not White? (Atewologun and Singh, 2010; Bhopal, 2010; Rodriguez and Scurry, 2019).

As you sit round the top table of the inner circle, you are unlikely to question the decisions of your colleagues, especially the central leader. If you should dare to challenge the status quo, you will be faced with a deadly silence at best.

As a Black woman, you are expected to be grateful to the liberals who fought for you, and to show gratitude. Any hint of assertiveness or constructive criticism is interpreted as hostility and is regarded as unacceptable and impolite in the extreme (Showunmi and Tomlin, 2022).

It is exceedingly difficult for Black women to gain a seat at the top table of the inner circle. It is also difficult for White women to rise to senior positions (Preston, 2019). There really is a glass ceiling even for White women. But that ceiling is a concrete ceiling if you are Black.

What does the glass or concrete ceiling represent? It is the obstacle you must smash in order to progress to top management (Sue et al., 2008). White women can see through the glass to the next step, and it is comparatively easy if dangerous to smash glass. But you can't even see-through concrete. You can't see the way forward and concrete is much harder and more impenetrable than glass. As a Black woman, you are hindered by more barriers and obstacles than your white counterparts. It's only when you become sufficiently emboldened to push your head up and try to approach the ceiling that you realize that you're getting a lot of bumps and bruises. You begin to appreciate that there is a huge mass in your way, preventing you from progressing (Showunmi and Tomlin, 2022).

As your White colleagues manage to smash the glass ceiling, you find yourself banging your head against the concrete time and time again. This saps your confidence and self-esteem. Discrimination is insidious. Irrespective of your ability and qualifications, it diverts you from your career path.

The culture in universities continues to favor the promotion of white men. Showunmi and Tomlin (2022) gathered data which illustrated how career progression for Black women academics was stalled because the focus was on preparing white males for senior positions:

...promoting white male colleagues and softening their fall or managing their mistakes, which is [a] completely different scenario for BME. Colleagues gathering round to mitigate and also providing mentoring/coaching to promote White males forms part of the organizational culture (Black female academic).

You may be lucky enough to be offered a helping hand that guides you and supports your progression. A respondent (Showunmi and Tomlin, 2022) commented: “You need to understand the environment you are placed in and to grasp the concept of self- awareness... Overcoming hurdles can be achieved with the right support.” (Black female academic).

But such backing will not be unconditional. You may be expected to remain unfailingly loyal to your champion, through thick and thin, whatever the circumstances. As a Black woman, you will need backing, because it is a myth that Black women and White women are subjected to the same degree of discrimination. It must be recognized that Black women face double discrimination on grounds of race as well as gender. Eradicating double discrimination is not an easy task because of the strength of prejudice and tradition.

Consider a Black woman who has been recently employed and wants to progress using the same approach as a White woman. This is where problems arise. As mentioned, Black women in England face an impenetrable concrete ceiling according to research undertaken by University of Manchester (2021).

There are more than 1.2 million Black and mixed-race women of Black heritage, many of whom work in diverse organizations in various roles. However, despite the many years of significant contributions made by Black women in the workplace, barriers to progression to a leadership position persist.

This is not only the case in the UK. Recently-published international data (McKinsey and Company, 2021) states: “At every step up the corporate ladder, women of color [sic] lose ground to White women and men of color”. At entry level, women of color represent 17% of the workforce but at Chief executive (C-suite) level the figure is 4% for women of color, but 20% for White wome (see https://www.fawcettsociety.org.uk/news/landmark-report-reveals-75-of-women-of-~colour-have-experienced-racism-at-work).

The intersection of sexism and racism constitutes an almost insurmountable obstacle (Lumby and Coleman, Fuller, 2013; Showunmi et al., 2016; Moorosi, 2021) which in itself perpetuates inequalities and prevents career advancement.

## Black women and leadership

Reviews at the intersection of leadership and diversity, such as Powell's (2012) often assume that groups of women are homogenous. Devine (1989) suggests that many Black stereotypes are transferred into perceptions of leadership. Understanding this enables us to make sense of the various studies that explore leadership. Whereas White leaders would be described as “working well under pressure” (Rudman and Glick, 2001, p. 751), “Black leaders are perceived as more incompetent than white leaders”.

Rudman and Glick's study of the experiences of Black professionals found that the challenges Black women face in the workplace are “real” and pervasive and that they result from complex layers of discrimination and inequality. There is ample evidence to demonstrate that Black leaders are always working against the negative images and stereotypes presented in the media (Collins, 2002; Gewertz, 2008; Osler and Webb, 2014).

Black leaders are subjected to double minority discrimination along with racial and/or gender-based microaggressions which “are the subtle verbal and nonverbal slights, insults, and disparaging messages directed toward an individual due to their gender, age, disability, and racial group membership often automatically and subconsciously” (Prieto et al., 2016, p. 36). Racial and/or gender-based microaggression is rooted in the conscious and subconscious reality of colonial racism that perpetuates the marginalization of unvalued others (Sue, 2010). It is one form of discrimination steeped in white supremacy that Black leaders experience in personal and professional situations. Despite seniority, a Black female professor confirmed the difficulties faced on a regular basis: “I think that I would have to say the challenges come daily, minutely in so many contexts” (Black Female professor) (Showunmi and Tomlin, 2022).

Research also suggests that the social identity group to which a leader belongs is considered a significant factor in leader effectiveness and the extent to which a leader may feel able to enact that identity (van Knippenberg et al., 2011). From a sociological perspective, this is explained by the extent to which the leader and the group see themselves as part of a collective or share the same social identity.

Showunmi's autobiographical account explored and examined a critical incident experienced as a newly appointed leader in a higher education establishment. The account probes the various challenges faced in a predominantly White institution. It reveals instances of invisibility being underpinned by low expectations, presumptions of not being academically able, and (White) surprise at a Black academic applying academic rigor in all aspects of their work. It further sheds light on the reasons for valuing the diverse theoretical frameworks of intersectionality, feminism as a way to open up opportunities for further debate and wider contributions to knowledge. Using the self as the starting point in the research was fundamental as the idea started with the lived experience of the researcher. Being able to critically engage with the lived experience of the research and then the participants provided an emerging hypothesis which was used as the next step in the research design process.

## Case study

The following case study illustrates how the theories in this paper are more than concepts. They are reflected in the lived experience of Black female leaders. The case details an academic leader's journey after acceptance of a post. This case study needs to be understood through a race and/or intersectional lens to completely grasp the complexity of the issues outlined. In addition, it is also necessary to acknowledge the Black female leader's disassociation of the pain and trauma caused by their experience of racial discrimination from White dysfunctional team members. Survival in a highly mono-cultural White environment would undoubtedly cause racial stress due to the reduced capacity of many Black academics to tolerate race-based stress or distress (lack of racial stamina). These defensive moves include physically removing oneself from the stress inducing situation, through for example internalization. This would result in denying or minimizing the continuing significance of race or of White privilege and sometimes becoming threatening and aggressive.

When reading the case study consider the following questions:

1) Why did the University hire Black leaders?
2) Was there any evidence that it was a positive action hire?

## Critical racial autobiographical case study

During 2008 I started work at an elite UK university as a senior manager. I was recruited to a department renowned for leadership development. However, the majority of the leaders were White staff and faculty and all of the Black staff and faculty except two, held positions of low responsibility.

(I was about to experience everyday racism) My role was to head up a section of five White members of staff and 20 associate staff members who were out in the field delivering executive education programmes. Most of the team consisted of retired Principals, Deputy Principals and Senior School Administrators.

When I received the notification that I had been appointed, I was ecstatic that I had been chosen to take on this enormous task. I can recall the very first day I asked the question, “Will there be an induction?” The response was: “You'd be lucky; ask the team members, they will induct you”, and then there was a laugh: “You are a senior member of staff after all”.

I would set up meetings to understand and establish the purpose of the work that my team were delivering. Prior to these meetings I had been aware that my new team was deemed “dysfunctional” and my appointment was to ensure that the individuals would start to work together as a team. My role was to make change happen. However, I had not been prepared for the rejection that lay ahead (self-blame and overt institutional racism). The team was not only resentful, but had not had a chance to prepare itself for a leader of my background. They clearly had in-built prejudices about the ability of a Black person to be a leader to them (White ignorance and microaggressions). Much of the resentfulness, in my opinion, could only be attributed to “race”. They made numerous criticisms about my ability to do the job which could not be substantiated. Working there was a miserable experience (microaggressions). Some of the White staff members isolated me, criticized me behind my back, refused to offer me any assistance, did not socialize with me, and attributed this to me being unsociable. They took no account of the fact that I was completely new to the role and made no attempt to accommodate me. They told me nothing about the work, and then criticized me for not knowing (institutionalized racism with violent behavior). I questioned and reflected again and again on whether I was to blame, and frequently changed my approach—but nothing changed. Eventually, after a difficult meeting with my team and senior management, I was given the option of moving to another department, which I accepted (White avoidance). Racism had won the day.

I was told by several managers both Black and White that I had handled the situation with dignity (gaslighting and/or White avoidance). I was left with unanswered questions though–what did that experience mean? I believe the experience was extremely insightful in terms of how institutional racism is enacted. It is these subtle and often unconscious processes that are operationalized which often cause some Black academics to leave the academy.

Given the information included in the critical racial autobiographical case study we need to consider the points raised and the way in which racial avoidance played out in the everyday experience of the appointed leader. An interviewee (Showunmi and Tomlin, 2022) illustrated the multiple challenges faced by Black female leaders in the academy:

I think you are prepared, as a Black person, if you are managing White people, to come across a couple of issues. I think what made it really interesting was the fact that Black people weren't receptive to me leading them as well. That came as a bit of a surprise (Black female academic).

The lack of recognition given to racial avoidance contributes to the hostile environment the leader struggles to navigate to achieve the intended goals of the newly appointed post. The recurring question which needs to be addressed is what triggered the micro-aggression toward the Black female leaders?^1^

## Conclusion

This review paper focuses on Black women's inequality in UK higher education and the workplace more broadly, adding to the literature. The paper starts with a brief analysis of what it means to be Black, explaining how terminology and evolving conceptions of “race” have contributed to the complex range of challenges faced by Black women in higher education and the workforce in general. Contemporary inequality is explored and everyday “sophisticated racism” (Showunmi and Tomlin, 2022) is outlined.

The work is timely, as it adopts a critical perspective on the many struggles of Black women, first to enter the workplace, then to prove their worth. Now they are getting through the door and sitting at the table, the challenge for organizations is how they can be valued and recognized for their brilliance. Their passion, energy, drive, and foresight are something that needs to be nurtured, not rejected, as organizations benefit from their engagement. This paper also studies the ways in which the many challenges faced by Black women and girls have been exacerbated by White women's insecurity, “White women syndrome” (Showunmi and Tomlin, 2022) and their reluctance to share the “equality” space.

This review paper illuminates and enriches what we already know and will stimulate critical and fruitful conversations on gender and race in the context of work. The significance of the dominance of White academics as authors of the literature on racism is highlighted. In the 1960s and 70s, the recognition and study of discrimination was emerging; it was inevitably White, mostly male writers who raised these issues. However, there has been a surge in the numbers of Black women researching the experiences of Black women and girls. The time has now come for White researchers to enable Black colleagues to shine, lending a helping hand where appropriate. Black women and girls need to be seen and heard instead of fading into the background. More research must be done to ensure that the inequality they are subjected to is thoroughly investigated.

Mental health, burnout, racial stress, and trauma is rife in the workplace. When Black women and girls seem to be coping, they may actually be suffering in silence. It is important to recognize that Black women and girls may also identify with a disability or belong to another group such as LGBTQI, or be affected by their age, class, or religious belief.

Few Black women and girls are aware that it is the challenges of multiple layers of discrimination which lead to the deterioration of their overall wellbeing. They are unable to recognize and articulate the experiences which underlie their unhappiness.

The conversation on racial trauma and its impact is just beginning to be understood. Senior leaders and board members alongside human resources and equality and diversity departments need to understand how this affects the overall performance of Black women in the workplace and take action to support them. Further research which engages with Black women and girls is needed as much remains to be explored to promote our understanding of inequality in the workplace. A closer examination of the workplace will shed light on the reasons for the failure of equality initiatives.

## Author contributions

The author confirms being the sole contributor of this work and has approved it for publication.

## Footnotes

^1^ https://assets.publishing.service.gov.uk/government/uploads/system/uploads/attachment_data/file/974507/20210331_-_CRED_Report_-_FINAL_-_Web_Accessible.pdf
